# Comparative Analysis of Kinematic and Identified MIMO Models in Model Predictive Control for Mobile Robot Trajectory Tracking

**DOI:** 10.3390/s26175431

**Published:** 2026-08-27

**Authors:** Diego Guffanti, Wilson Pavon, Alfredo Zapata

**Affiliations:** 1 Facultad de Ciencias de la Ingeniería e Industrias, Universidad UTE, Quito 170129, Ecuador; wilson.pavon@ute.edu.ec; 2 Departamento de Ciencias Exactas, Universidad de las Fuerzas Armadas ESPE, Sangolquí 171103, Ecuador; hazapata@espe.edu.ec

**Keywords:** Model Predictive Control (MPC), trajectory tracking, predictive model, system identification, differential-drive mobile robot

## Abstract

Trajectory tracking remains one of the main challenges in mobile robotics, particularly when robots operate under real-world disturbances and modeling uncertainties. Model Predictive Control (MPC) has become one of the most effective solutions for this problem because of its ability to optimize future control actions while explicitly handling system constraints. However, despite the variety of predictive models reported in the literature, there is still limited experimental evidence regarding how predictive-model selection influences the closed-loop behavior of mobile robots. This work presents an experimental comparison between two MPC implementations: a Kinematic MPC and a multiple-input multiple-output MPC (MIMO-MPC) based on an experimentally identified state-space model. Both controllers were implemented on the same four-wheel differential-drive mobile robot running Robot Operating System 2 (ROS 2) and were configured with identical prediction horizons, weighting matrices, constraints, reference trajectories, and disturbance sequences, enabling a direct experimental comparison of the influence of predictive-model selection on closed-loop performance. Performance was assessed through trajectory-tracking accuracy, disturbance recovery, and control smoothness metrics under both nominal and externally perturbed operating conditions. The experimental evaluation was performed on real hardware at 50 Hz using a closed-loop trajectory of approximately 20 m. Under nominal conditions, the kinematic MPC achieved a lower lateral root mean square error (RMSE) (0.1031 m versus 0.1187 m) and maintained 93.23% of the trajectory within the ±0.20 m tolerance band. Under external perturbations, however, the MIMO-MPC reduced the heading RMSE from 0.7617 rad to 0.6503 rad, shortened the recovery time after the two largest disturbances by up to 35.6%, and decreased the angular-velocity rate root mean square (RateRMS) and jerk root mean square (JerkRMS) by approximately 24.7% and 24.8%, respectively. These results demonstrate that predictive-model selection has a significant influence on the transient behavior of MPC. While a conventional kinematic model provides excellent nominal tracking performance, an experimentally identified MIMO predictive model offers faster recovery and smoother control under disturbed conditions. The proposed experimental methodology provides practical evidence that can assist the selection of predictive models for MPC-based mobile robots according to the expected operating conditions.

## 1. Introduction

Trajectory tracking is one of the fundamental problems in autonomous mobile robotics, particularly for differential-drive mobile robots (DDMRs) operating in structured and semi-structured environments. Accurate path following is essential in numerous applications, including warehouse automation, service robotics, autonomous inspection, and precision agriculture, where tracking errors directly affect navigation safety and task execution. Among the available control strategies, Model Predictive Control (MPC) has become one of the most widely adopted approaches because it explicitly incorporates system constraints while optimizing future control actions over a finite prediction horizon. Owing to these characteristics, MPC has demonstrated excellent trajectory-tracking capabilities in both simulation and real robotic platforms [1].

Recent research has focused on improving different aspects of MPC for mobile robots. Xu et al. [2] proposed a Dynamic Model Predictive Control (DMPC) for a three-wheeled independent drive and steering mobile robot, demonstrating improved tracking accuracy on highly curved trajectories. Ali et al. [3] incorporated Control Barrier Functions into MPC to guarantee obstacle avoidance while preserving trajectory-following performance. Abed et al. [4] introduced a nonlinear neural-network fractional controller to compensate for modeling uncertainties, whereas Zhang et al. [5] reviewed recent learning-based MPC techniques capable of improving trajectory tracking under uncertain operating conditions. More recently, Tang et al. [6] demonstrated that data-driven predictive models based on deep Koopman operators can significantly improve tracking performance when the system dynamics become difficult to model analytically. These studies illustrate the continuing effort to improve the predictive capability of MPC through more accurate system representations.

An equally important research direction concerns the selection of the predictive model itself. Most MPC implementations for DDMRs employ simplified kinematic models because of their low computational cost and satisfactory performance for real-time navigation. Alternatively, several researchers have proposed dynamic or experimentally identified models to capture actuator dynamics, wheel–ground interaction, and other non-ideal behaviors that cannot be represented by purely kinematic equations. For example, Bouzoualegh et al. [7] proposed a dynamic-model-based MPC for a differential-drive mobile robot, while Mondal et al. [8] compared kinematic and dynamic predictive models for trajectory tracking. However, these studies generally modify not only the predictive model but also other elements of the controller, such as the optimization formulation, controller tuning, or disturbance-compensation strategy. Consequently, the individual contribution of the predictive model to the overall closed-loop performance remains difficult to quantify experimentally.

Therefore, despite the extensive literature on MPC for mobile robots, there is still limited experimental evidence in which the predictive model is isolated as the only varying component while all remaining controller parameters are kept identical. Therefore, the specific influence of model fidelity on closed-loop trajectory-tracking performance, disturbance recovery, and control smoothness has not yet been thoroughly quantified through real-world experiments using differential-drive mobile robots.

To address this gap, this paper presents an experimental comparison between two MPC implementations that differ exclusively in their predictive model. The first controller corresponds to a Kinematic MPC, whereas the second employs a multiple-input multiple-output (MIMO) MPC based on an experimentally identified state-space model. Both controllers share the same prediction horizon, optimization problem, weighting matrices, constraints, sampling period, reference trajectory, and disturbance sequence. Their performance is evaluated through real experiments conducted under nominal operating conditions and under externally applied perturbations using trajectory-tracking accuracy, recovery time, and control smoothness metrics.

The main contributions of this work are summarized as follows:

- An experimental comparison between a Kinematic MPC and a MIMO-MPC, enabling the isolated influence of predictive-model fidelity on closed-loop trajectory-tracking performance to be evaluated.
- The implementation and validation of an experimentally identified MIMO state-space model integrated as the predictive model of an MPC controller for a four-wheel differential-drive mobile robot operating under Robot Operating System 2 (ROS 2).
- A comprehensive real-world experimental evaluation under both nominal and externally perturbed operating conditions using trajectory-tracking accuracy, disturbance recovery, and control smoothness metrics.
- An experimental assessment of the influence of predictive-model selection on the performance of MPC for differential-drive mobile robots, providing practical insights into the trade-offs between conventional kinematic and experimentally identified predictive models.

The remainder of this paper is organized as follows. Section 2 reviews the state of the art in MPC for mobile robots and predictive modeling. Section 3 describes the experimental platform, the system identification procedure, and the two MPC controllers. Section 4 presents the results of the experimental evaluation. Section 5 discusses the main findings and their implications. Finally, Section 6 summarizes the main conclusions and outlines future research directions.

## 2. State of the Art

Trajectory tracking in differential-drive mobile robots has motivated extensive research on predictive control techniques capable of optimizing future control actions while satisfying actuator and motion constraints. Among these techniques, Model Predictive Control (MPC) has become one of the most widely adopted frameworks because its performance depends directly on the predictive model used to estimate the future behavior of the robot. The theoretical foundations summarized by Mayne et al. [1] established the conditions under which constrained MPC can provide feasible and stable closed-loop behavior. Since then, MPC has been applied to different mobile platforms using linear, nonlinear, kinematic, and dynamic formulations. For wheeled mobile robots, Yang et al. [9] proposed an MPC scheme based on a linearized kinematic tracking-error model with softened constraints and limits on both the control inputs and their increments. Their results showed that a comparatively simple predictive model can provide accurate and smooth tracking when combined with an appropriate optimization formulation. More recently, Ali et al. [3] combined linear MPC, dynamic feedback linearization, and Control Barrier Functions for differential-drive robots, illustrating how simplified predictive formulations can also be extended to address safety and obstacle-avoidance requirements.

A central design decision in MPC is the selection of the model used to predict the future behavior of the controlled system. Kinematic models are frequently adopted for differential-drive mobile robots because they provide a compact representation of the non-holonomic motion and can be evaluated efficiently in real time. Alternatively, dynamic models can represent effects associated with actuator response, inertia, tire or wheel interaction, and other behaviors that are not explicitly included in ideal kinematic equations. Bouzoualegh et al. [7] formulated an MPC for a differential-drive robot using a nonlinear dynamic model transformed through input–output linearization. However, their evaluation was conducted in simulation and did not compare the dynamic formulation against an otherwise identical kinematic MPC. Mondal et al. [8] more directly studied the trade-offs between kinematic- and dynamic-model-based linear MPC for a non-holonomic robot. Their dynamic formulation incorporated the bandwidth limitations of an inner velocity-control loop, and the study examined when the simpler kinematic approximation was sufficient and when the additional dynamic representation became relevant. Nevertheless, the compared formulations were embedded in a hierarchical control architecture, making it difficult to attribute the observed differences exclusively to the predictive model itself.

The influence of model selection has also been investigated extensively in autonomous vehicle control. Falcone et al. [10] compared nonlinear and successively linearized vehicle models within an MPC-based active-steering framework and analyzed the trade-off between prediction accuracy and computational complexity. Their controller was validated through simulations and experiments on icy roads, demonstrating that model formulation is closely related to both real-time feasibility and closed-loop behavior. Although road vehicles and differential-drive robots exhibit different dynamics and operating ranges, this work established an important methodological precedent: the predictive model should be selected by considering not only its physical fidelity but also the computational requirements of online optimization. However, such studies do not directly determine whether an experimentally identified state-space model improves the performance of a low-speed differential-drive robot when all the remaining controller parameters are held constant.

A second body of research improves MPC robustness by augmenting the nominal predictive model with explicit disturbance-handling mechanisms. Sun et al. [11] combined disturbance observers with MPC to address matched disturbances in non-holonomic mobile robots, while Sun et al. [12] developed tube-based and nominal robust MPC formulations for constrained unicycle robots subjected to bounded additive disturbances. Li et al. [13] subsequently incorporated disturbance compensation into MPC for wheeled mobile robots navigating in environments with obstacles. Similarly, Wang et al. [14] integrated an adaptive sliding-mode observer with MPC to estimate lumped disturbances and model uncertainties in a Mecanum-wheeled robot. These approaches improve robustness by combining the predictive controller with observers, constraint tightening, ancillary feedback, or feedforward compensation. Consequently, their reported performance reflects the combined contribution of several control components rather than the isolated effect of replacing the predictive model.

Data-driven modeling has recently emerged as another means of increasing prediction fidelity. Tang et al. [6] constructed a linear predictive representation of a wheeled mobile robot using a deep Koopman operator and combined it with an extended-state observer, adaptive prediction horizon, and self-triggered MPC. Their simulation results showed a substantial reduction in tracking error relative to the selected conventional MPC benchmark. This work demonstrates the potential of learned predictive representations, but the simultaneous incorporation of disturbance estimation and adaptive optimization makes it difficult to determine how much of the improvement originates exclusively from the learned model. Experimental system identification offers a more direct alternative in which a state-space model is obtained from the measured relationship between the commands and the actual robot response; however, controlled experimental comparisons between such models and conventional kinematic representations remain limited.

Large deviations from the reference trajectory have also motivated strategies that act beyond the nominal prediction model. Guffanti et al. [15] introduced a dynamic look-ahead re-entry strategy that temporarily reshapes the local reference when a differential-drive robot exits a prescribed lateral tolerance band. Real-robot experiments showed that an additional recovery mechanism can improve robustness under deliberately applied perturbations. Nevertheless, this approach changes the reference-generation strategy while retaining the underlying MPC structure. It therefore addresses a different but complementary question to the one considered in the present study: whether the post-disturbance behavior can be improved by changing only the predictive model, without introducing observers, robust tubes, disturbance estimators, adaptive horizons, or explicit re-entry mechanisms.

In summary, the literature has investigated kinematic and dynamic MPC formulations, disturbance-rejection and robust MPC, observer-based compensation, safety-aware control, and data-driven predictive modeling. These studies demonstrate that both model fidelity and auxiliary control mechanisms can influence trajectory-tracking performance. However, real-world evidence remains limited for comparisons in which the predictive model is isolated as the only varying component. The present work addresses this gap by experimentally comparing a conventional kinematic model and an identified MIMO state-space model within the same MPC framework. Both implementations use identical sampling periods, prediction horizons, cost weights, constraints, reference trajectories, and disturbance sequences, allowing the influence of predictive-model selection on tracking accuracy, disturbance recovery, and control smoothness to be evaluated independently.

## 3. Methods

### 3.1. Mobile Robotic Platform

**Figure 1:**
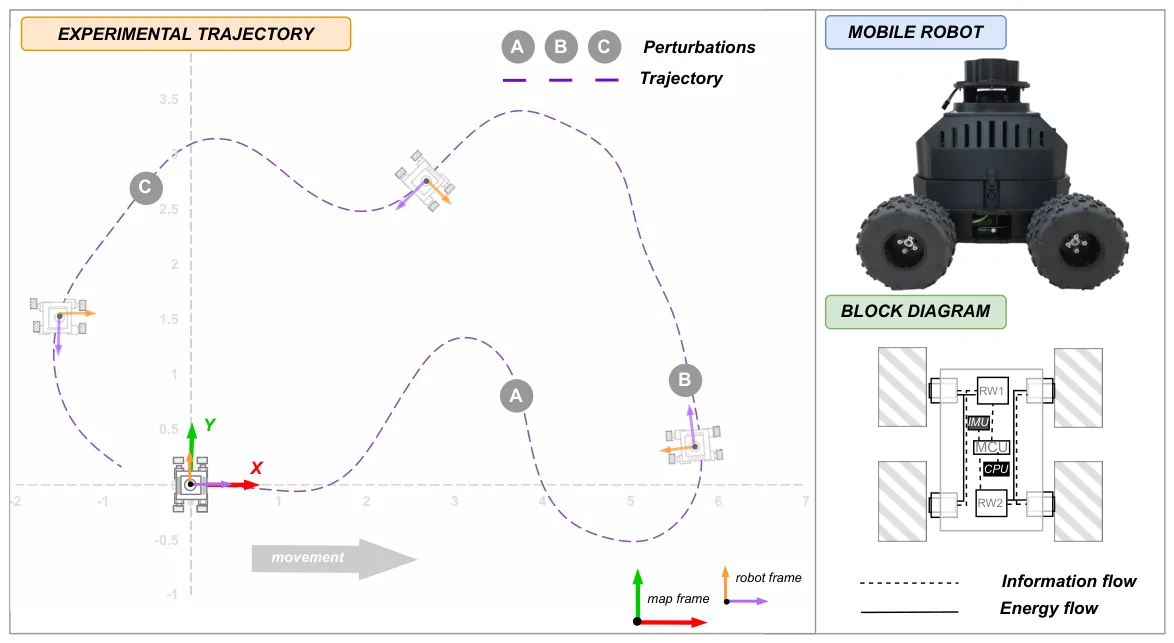
Differential-drive mobile robot platform and experimental environment.

The experimental platform used in this study is a custom-built four-wheel differential-drive mobile robot designed for indoor navigation experiments. The robot is mounted on a Recon Chassis Kit, with the left and right wheel sets independently actuated. Figure 1 shows the mobile platform, its main hardware components, and the experimental trajectory. The principal mechanical, sensing, electronic, and computational specifications of the platform are summarized in Table 1.

The robot is driven by four planetary gear motors equipped with integrated quadrature encoders. Motor actuation is handled by two dual-channel ROBOCLAW motor controllers (RW1 and RW2 in Figure 1), allowing independent control of the left and right wheel sets. Encoder feedback is used to measure wheel angular velocities and estimate robot motion.

The sensing system comprises the wheel encoders, an inertial measurement unit (IMU), and a 2D LiDAR sensor. The IMU provides angular velocity and linear acceleration measurements, while the LiDAR provides environmental information for localization. Low-level hardware interfacing is performed by an STM32 NUCLEO-F207ZG microcontroller (MCU in Figure 1), which communicates with the motor controllers through a USART serial interface. Encoder and IMU data are transmitted to the onboard computer through a USB serial connection.

High-level computation is performed by an Intel NUC onboard computer (CPU in Figure 1) running Ubuntu 20.04 and ROS 2 Foxy Fitzroy. A dedicated high-capacity power bank supplies the onboard computer, while a 4S lithium battery powers the motors, motor controllers, microcontroller, and sensors. Robot localization and odometry estimation are performed within the ROS 2 ecosystem using Nav2 and SLAM Toolbox. Wheel encoder, IMU, and LiDAR measurements are used for state estimation and localization, and the resulting pose estimate is recorded during the experimental trials. All system components operate with a common sampling period of $T_{s}=0.0202\;s$ (50 Hz), ensuring synchronized sensing and actuation throughout the experiments.

### 3.2. Identification of the MIMO State-Space Model

The dynamic behavior of the mobile robot is modeled as a multiple-input multiple-output (MIMO) system due to the coupled relationship between its commanded and measured velocities. The system inputs are the reference linear and angular velocities, denoted as $v_{ref}\left(t\right)$ and $\omega_{ref}\left(t\right)$, while the outputs correspond to the actual robot velocities v(t) and ω(t). Although the ideal kinematic model of a differential-drive robot assumes decoupled dynamics, real robotic platforms may exhibit coupling effects arising from actuator dynamics, internal velocity control loops, friction, and mechanical asymmetries. For this reason, an experimentally identified MIMO state-space model is obtained using a data-driven system identification approach.

The identification process is based on experimental data collected during a continuous 5.48-minute experiment in which the robot was manually driven using a radio control (RC) system. This procedure ensured sufficiently rich excitation of both linear and angular velocity channels across a wide operating range, including repeated accelerations, decelerations, starts, stops, changes in direction, and turning maneuvers at different linear and angular velocities. During the experiment, the reference inputs $v_{\mathrm{ref}}\left(t\right)$ and $\omega_{\mathrm{ref}}\left(t\right)$, together with the corresponding measured robot velocities v(t) and ω(t), were recorded with a sampling period of $T_{s}=0.0202$ s, resulting in a total of 16,293 samples.

Since the identification data were collected directly from the physical robot, the measured signals inherently include sensor noise and experimental variability associated with real-world operation. Therefore, model identification and validation were performed using the measured signals rather than idealized or simulated data.

The dataset was subsequently split into two subsets: 90% of the samples (14,663 samples) were used for model estimation, while the remaining 10% (1630 samples) were reserved for validation.

The state-space model parameters were estimated using a prediction-error minimization (PEM) method initialized through subspace identification, as implemented by the ssest function of the MATLAB R2023b System Identification Toolbox. The method first obtains an initial state-space model using a subspace identification approach and subsequently refines the model parameters through iterative prediction-error minimization. The continuous-time state-space matrices *A*, *B*, *C*, and *D* were estimated from the training dataset for each candidate model order. Models with orders from 1 to 5 were estimated using the same procedure and subsequently compared using the independent validation dataset.

To determine the appropriate complexity of the model, state-space models with orders ranging from 1 to 5 were identified and evaluated using the validation dataset. The prediction quality of each model was assessed using the FIT and RMSE metrics for both the linear and angular velocity outputs. Table 2 summarizes the results obtained.

The first-order model fails to adequately represent the angular velocity dynamics, as indicated by a negative FIT value and a large RMSE. Increasing the model order from one to two leads to a substantial improvement in prediction accuracy for both velocity channels. Although third- and fourth-order models achieve slightly higher FIT values, particularly for the linear velocity, the performance gains with respect to the second-order model are marginal. Moreover, these higher-order models introduce additional states, increasing model complexity without a commensurate improvement in accuracy. The fifth-order model exhibits a degradation in angular velocity performance, suggesting overfitting effects. Based on this trade-off between predictive accuracy and model simplicity, the second-order model is selected for the remainder of this study.

**Figure 2:**
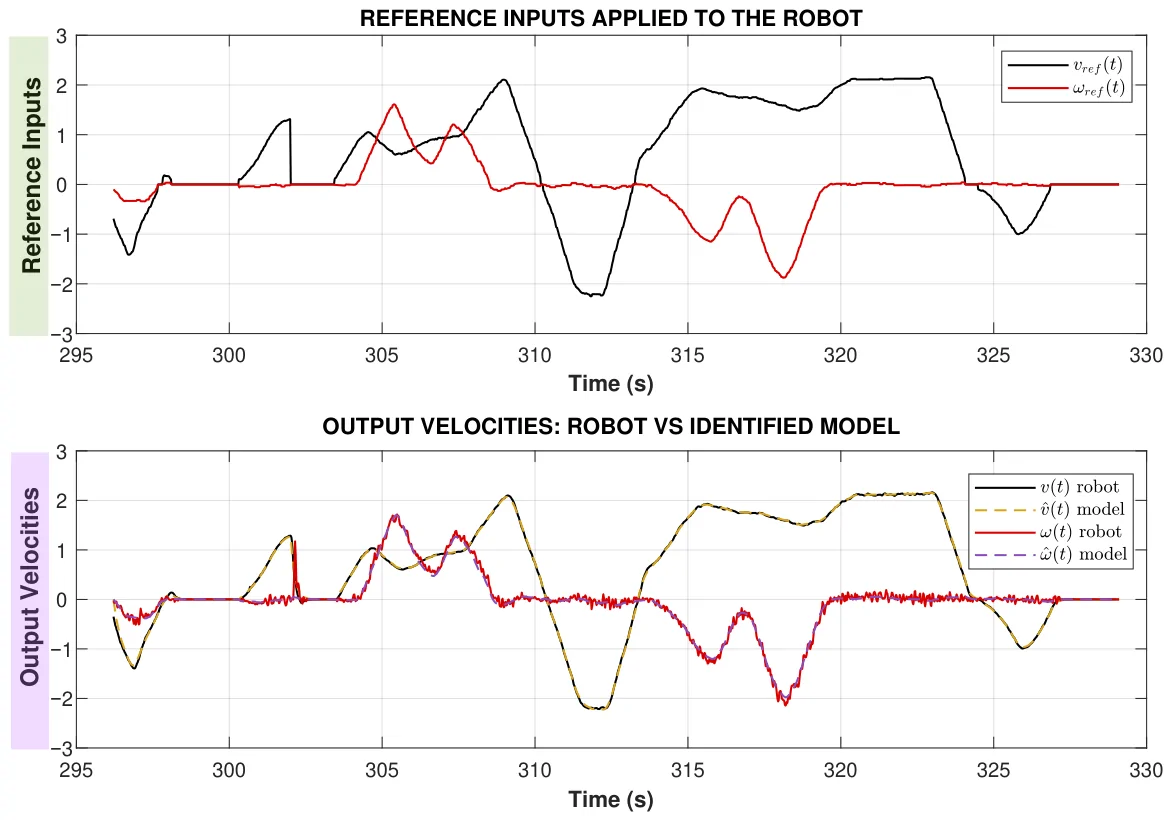
Time-domain validation of the experimentally identified second-order MIMO state-space model. The reference inputs $v_{ref}\left(t\right)$ and $\omega_{ref}\left(t\right)$ applied to the robot are shown in the upper subplot. The lower subplot compares the measured robot velocities v(t) and ω(t) with the corresponding model outputs $\hat{v}\left(t\right)$ and $\hat{\omega }\left(t\right)$ obtained from the identified model.

After selecting the model order, the identified second-order MIMO model is validated in the time domain. Figure 2 presents a representative segment of the validation dataset. The upper subplot shows the applied reference inputs $v_{ref}\left(t\right)$ and $\omega_{ref}\left(t\right)$, while the lower subplot compares the measured robot velocities v(t) and ω(t) with the corresponding model outputs $\hat{v}\left(t\right)$ and $\hat{\omega }\left(t\right)$. The close agreement between measured and estimated velocities across different motion regimes demonstrates that the selected model accurately captures the dominant transient and steady-state dynamics of the robot.

**Figure 3:**
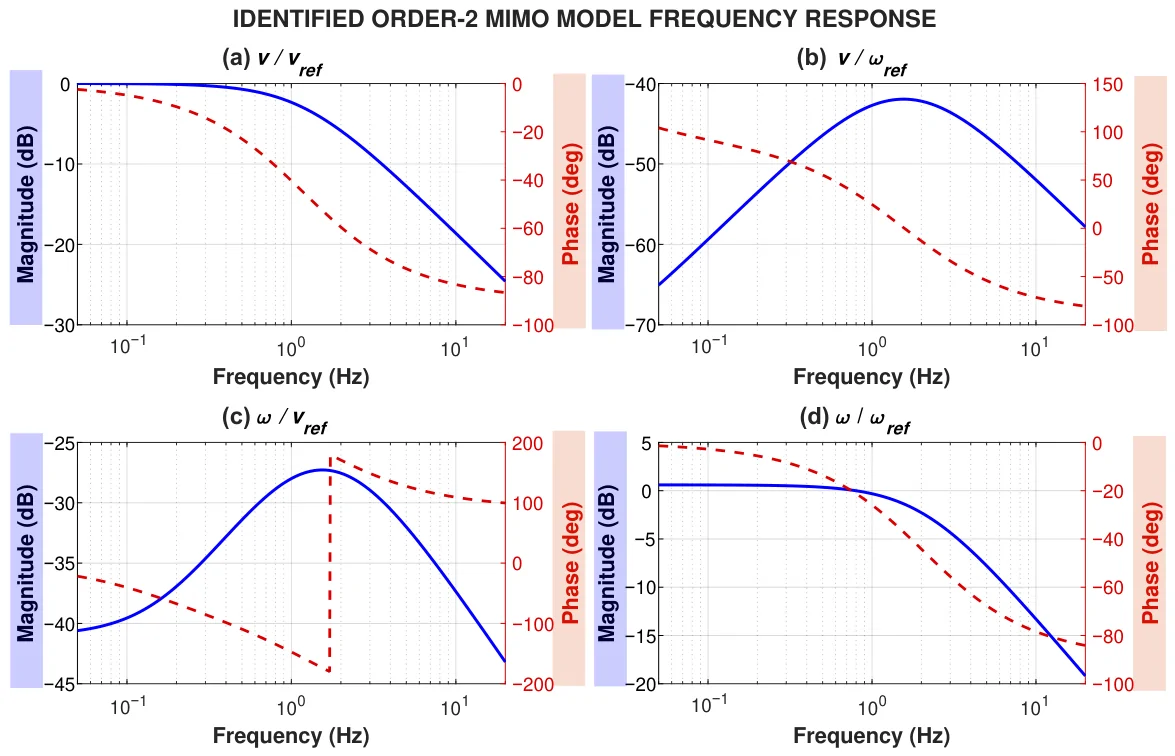
Frequency-domain representation of the experimentally identified second-order MIMO state-space model: (**a**) $v\left(s\right)/v_{ref}\left(s\right)$, (**b**) $v\left(s\right)/\omega_{ref}\left(s\right)$, (**c**) $\omega \left(s\right)/v_{ref}\left(s\right)$, and (**d**) $\omega \left(s\right)/\omega_{ref}\left(s\right)$. Blue solid lines represent magnitude (dB) and red dashed lines represent phase (deg).

A frequency-domain analysis is performed to further examine the identified dynamics and reveal coupling effects between the input–output channels. Figure 3 shows the Bode magnitude and phase plots of the identified MIMO model. The direct channels $v\left(s\right)/v_{ref}\left(s\right)$ and $\omega \left(s\right)/\omega_{ref}\left(s\right)$ exhibit a low-pass behavior, which is consistent with the dynamics of the motors, drivers, and embedded velocity control loops. In contrast, the cross-coupling channels $v\left(s\right)/\omega_{ref}\left(s\right)$ and $\omega \left(s\right)/v_{ref}\left(s\right)$ present significantly lower magnitudes, typically below −40dB at low frequencies. This indicates weak but non-negligible coupling between linear and angular motions, which cannot be captured by a purely decoupled model. The smooth phase evolution across the frequency range suggests physically consistent secondary dynamics rather than identification artifacts.

The identified continuous-time MIMO state-space model is expressed as

(1) $$\dot{z}\left(t\right)=Az\left(t\right)+Bu\left(t\right),\;y\left(t\right)=Cz\left(t\right)+Du\left(t\right),$$

where $u\left(t\right)={\left[v_{ref}\left(t\right)\;\;\omega_{ref}\left(t\right)\right]}^{⊤}$ and $y\left(t\right)={\left[v\left(t\right)\;\;\omega \left(t\right)\right]}^{⊤}$. The identified system matrices are given by

(2) $$\begin{array}{cc} A & =\left[\begin{array}{cc} -7.374 & 0.3149\\ -0.9801 & -12.97 \end{array}\right],\;B=\left[\begin{array}{cc} 0.7284 & 0.03463\\ 0.02824 & -0.992 \end{array}\right],\\ C & =\left[\begin{array}{cc} 10.18 & 0.1914\\ -0.659 & -14.01 \end{array}\right],\;D=\left[\begin{array}{cc} 0 & 0\\ 0 & 0 \end{array}\right]. \end{array}$$

For implementation purposes, the model is discretized using a zero-order hold with sampling period $T_{s}=0.0202\;s$, yielding

(3) $$z_{k+1}=A_{d}z_{k}+B_{d}u_{k},\;y_{k}=C_{d}z_{k}+D_{d}u_{k}.$$

with

(4) $$\begin{array}{cc} A_{d} & =\left[\begin{array}{cc} 0.8616 & 0.0052\\ -0.0161 & 0.7695 \end{array}\right],\;B_{d}=\left[\begin{array}{cc} 0.0137 & 0.0006\\ 0.0004 & -0.0176 \end{array}\right],\\ C_{d} & =\left[\begin{array}{cc} 10.18 & 0.1914\\ -0.659 & -14.01 \end{array}\right],\;D_{d}=\left[\begin{array}{cc} 0 & 0\\ 0 & 0 \end{array}\right]. \end{array}$$

This discrete-time representation is subsequently employed as the internal prediction model within the model predictive control framework presented in the following section.

### 3.3. Model Predictive Control Formulation

Two MPC strategies are implemented and compared: a Kinematic MPC and a MIMO-MPC. The Kinematic MPC employs a conventional kinematic model, whereas the MIMO-MPC employs an experimentally identified MIMO state-space model integrated with an augmented pose–velocity prediction model. In both cases, the optimization variable is the stacked reference-input sequence

(5) $$U={\left[u_{0}^{⊤}\;u_{1}^{⊤}\;\ldots \;u_{N_{p}-1}^{⊤}\right]}^{⊤}\in R^{2N_{p}},\;u_{k}={\left[v_{\mathrm{ref},k}\;\;\omega_{\mathrm{ref},k}\right]}^{⊤}\in R^{2},$$

subject to the box constraints $U_{\min}\leq U\leq U_{\max}$. These constraints are imposed directly on the optimization variable at every prediction step, such that the QP solver searches for the optimal control sequence only within the feasible input region. In particular, the bounds limit the reference linear and angular velocity commands $v_{\mathrm{ref},k}$ and $\omega_{\mathrm{ref},k}$ throughout the prediction horizon. Consequently, the first control action extracted from the optimal sequence inherently satisfies the prescribed actuator constraints. The tracking objective is defined in pose coordinates using the reference states

(6) $$x_{i}^{\mathrm{ref}}={\left[x_{i}^{\mathrm{ref}}\;y_{i}^{\mathrm{ref}}\;\theta_{i}^{\mathrm{ref}}\right]}^{⊤},\;i=0,\ldots ,N_{p}-1,$$

constructed from the global trajectory by selecting the nearest reference point, extending forward until a fixed spatial horizon $L_{\mathrm{horiz}}$ is reached, and uniformly resampling the resulting segment into exactly $N_{p}$ reference states.

#### 3.3.1. Selection of $N_{p}$ and $L_{\mathrm{horiz}}$

**Figure 4:**
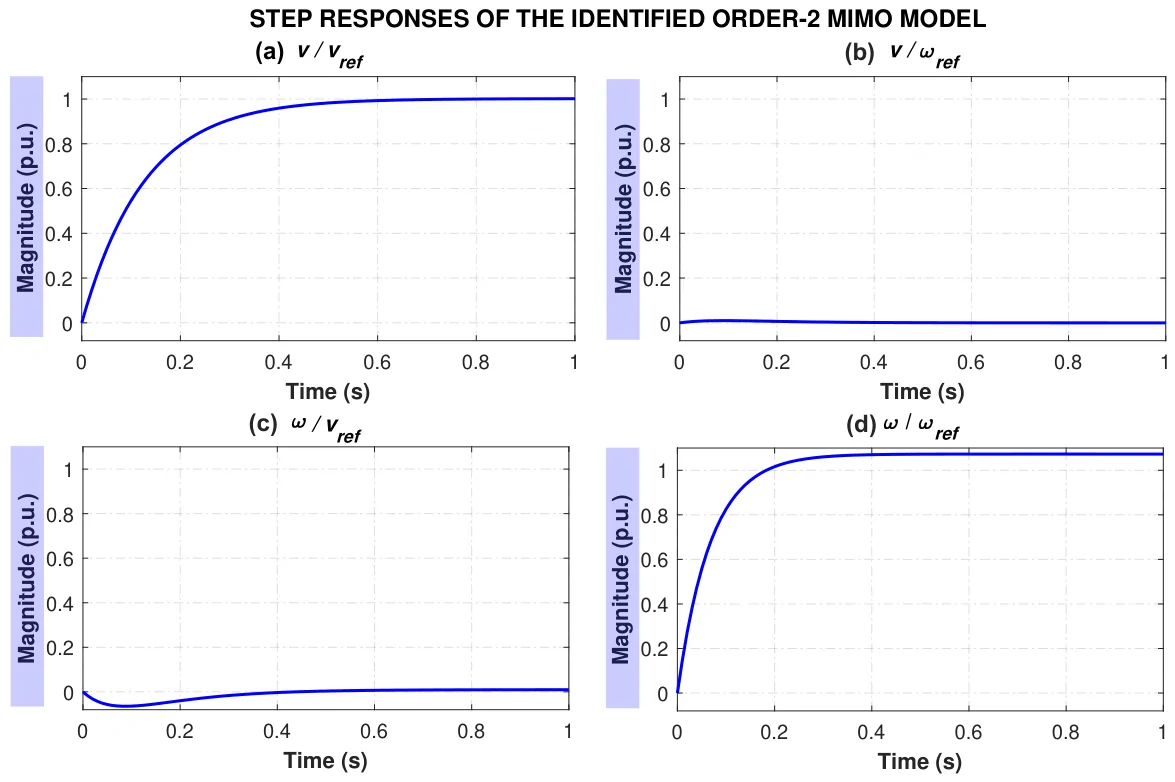
Time -domain representation of the experimentally identified second-order MIMO state-space model: (**a**) $v\left(t\right)/v_{\mathrm{ref}}\left(t\right)$, (**b**) $v\left(t\right)/\omega_{\mathrm{ref}}\left(t\right)$, (**c**) $\omega \left(t\right)/v_{\mathrm{ref}}\left(t\right)$, and (**d**) $\omega \left(t\right)/\omega_{\mathrm{ref}}\left(t\right)$. Step response curves are shown in blue solid lines for all input–output channels.

The prediction horizon $N_{p}$ is selected to capture the dominant velocity dynamics of the experimentally identified MIMO model. Let $T_{s}$ denote the sampling period and $T_{\mathrm{set}}$ the settling time estimated from the normalized step responses of the identified velocity model (Figure 4). The prediction horizon is chosen according to

(7) $$N_{p}\geq \left\lceil \frac{T_{\mathrm{set}}}{T_{s}}\right\rceil ,$$

so that the prediction horizon spans the dominant transient dynamics. Using $T_{s}=0.02\;s$ and $T_{\mathrm{set}}\approx 0.30\;s$ from Figure 4, Equation (7) yields

(8) $$N_{p}\approx \left\lceil \frac{0.30}{0.02}\right\rceil =15.$$

The spatial horizon was set to $L_{\mathrm{horiz}}=1.0\;m$, providing sufficient preview of the reference trajectory while maintaining reliable prediction accuracy during trajectory tracking.

To express tracking errors in a trajectory-aligned coordinate frame, the planar position error is rotated using a local reference transformation. Let $\psi_{i}$ denote the heading of the reference trajectory at prediction step *i*, defined as $\psi_{i}=\theta_{i}^{\mathrm{ref}}$. The corresponding rotation matrix is

(9) $$R\left(\psi_{i}\right)=\left[\begin{array}{cc} \cos\psi_{i} & \sin\psi_{i}\\ -\sin\psi_{i} & \cos\psi_{i} \end{array}\right],$$

which transforms position errors from the global frame into a local coordinate frame aligned with the tangent direction of the reference trajectory.

#### 3.3.2. Kinematic MPC (Baseline)

The Kinematic MPC employs the discrete-time kinematic model

(10) $$\begin{array}{c} x_{k+1}=x_{k}+T_{s}v_{\mathrm{ref},k}\cos\theta_{k},\\ y_{k+1}=y_{k}+T_{s}v_{\mathrm{ref},k}\sin\theta_{k},\\ \theta_{k+1}=\theta_{k}+T_{s}\omega_{\mathrm{ref},k}, \end{array}$$

where $u_{k}={\left[v_{\mathrm{ref},k}\;\omega_{\mathrm{ref},k}\right]}^{⊤}$.

The Kinematic MPC is intentionally formulated as a simplified baseline and does not explicitly model skid-steer dynamic effects such as wheel slip, lateral interaction forces, or possible center-of-mass offsets. Instead, the local kinematic prediction is updated at every control iteration using the current robot orientation $\theta_{k}$. Consequently, the coefficients associated with the translational motion depend on $\cos\theta_{k}$ and $\sin\theta_{k}$ and are recomputed at each sampling instant rather than remaining fixed throughout the experiment. The reference velocity commands $v_{\mathrm{ref},k}$ and $\omega_{\mathrm{ref},k}$ remain optimization inputs and therefore do not explicitly parameterize these local kinematic coefficients. This simplified formulation is deliberately retained as the baseline against which the experimentally identified velocity dynamics incorporated into the MIMO-MPC are evaluated.

Using a local kinematic approximation evaluated at the current robot orientation yields the lifted prediction model

(11) $$X=\Phi x_{k}+\Gamma U,\;X\in R^{3N_{p}},$$

where *X* stacks the predicted robot poses over the prediction horizon. The quadratic objective function is

(12) $$J=\sum_{i=0}^{N_{p}-1}∥e_{i}{∥}_{Q}^{2}+\sum_{i=0}^{N_{p}-1}{∥u_{i}∥}_{R}^{2},$$

where $Q\in R^{3\times 3}$ penalizes the pose tracking error and $R\in R^{2\times 2}$ penalizes the control effort. The stacked weighting matrices are

(13) $$Q_{\mathrm{big}}=\mathrm{blkdiag}\left(Q,\ldots ,Q_{f}\right)\in R^{3N_{p}\times 3N_{p}},\;R_{\mathrm{big}}=I_{N_{p}}⊗R\in R^{2N_{p}\times 2N_{p}},$$

where $Q_{f}$ is an optional terminal weighting matrix. Defining the block-diagonal transformation

(14) $$T=\mathrm{blkdiag}(T_{0},\ldots ,T_{N_{p}-1}),$$

with

(15) $$T_{i}=\left[\begin{array}{cc} R(\psi_{i}) & 0\\ 0 & 1 \end{array}\right]\in R^{3\times 3},$$

the optimization problem is formulated as

(16) $$\begin{array}{cccc} \min_{U}\; & \frac{1}{2}U^{⊤}HU+f^{⊤}U,\;H & =\Gamma^{⊤}T^{⊤}Q_{\mathrm{big}}T\Gamma +R_{\mathrm{big}},\;f & =\Gamma^{⊤}T^{⊤}Q_{\mathrm{big}}\left(T\Phi x_{k}-TX^{\mathrm{ref}}\right) \end{array}$$

The complete MPC (Baseline) formulation for the Differential-Drive Robot is summarized in Algorithm 1.
**Algorithm 1:** Kinematic MPC (Baseline) for Differential-Drive Robot**Require:** 
Sampling time $T_{s}$, prediction horizon $N_{p}$, spatial horizon $L_{\mathrm{horiz}}$, weighting matrices Q⪰0, R≻0, input bounds $U_{\min},U_{\max}$, reference path P1:**while** goal not reached **do**2:    Read current state $x_{k}={\left[x_{k},y_{k},\theta_{k}\right]}^{⊤}$3:    Find the nearest reference point on P4:    Construct the reference horizon ${\left\{x_{i}^{\mathrm{ref}}\right\}}_{i=0}^{N_{p}-1}$ by accumulating arc length up to $L_{\mathrm{horiz}}$ and uniformly resampling the resulting segment into $N_{p}$ states5:    Update the local kinematic approximation using the current orientation $\theta_{k}$6:    Construct the lifted prediction matrices (Φ,Γ)7:    Construct $Q_{\mathrm{big}}=\mathrm{blkdiag}\left(Q,\ldots ,Q_{f}\right)$8:    Construct $R_{\mathrm{big}}=I_{N_{p}}⊗R$9:    Construct the transformation matrix *T*10:  Compute $H=\Gamma^{⊤}T^{⊤}Q_{\mathrm{big}}T\Gamma +R_{\mathrm{big}}$11:  Compute $f=\Gamma^{⊤}T^{⊤}Q_{\mathrm{big}}\left(T\Phi x_{k}-TX^{\mathrm{ref}}\right)$12:  Solve $\min_{U}\frac{1}{2}U^{⊤}HU+f^{⊤}U$13:         subject to $U_{\min}\leq U\leq U_{\max}$14:    Apply the first optimal control input $u_{k}^{★}={\left[v_{\mathrm{ref},k}^{★},\;\omega_{\mathrm{ref},k}^{★}\right]}^{⊤}$

#### 3.3.3. MIMO-MPC with Augmented Pose Prediction

The MIMO-MPC employs an experimentally identified MIMO state-space model together with an explicit pose prediction, allowing the identified velocity dynamics of the robot to be incorporated while penalizing trajectory-tracking errors in Cartesian space. The complete receding-horizon optimization procedure executed at each sampling instant is summarized in Algorithm 2.
**Algorithm 2:** MIMO-based MPC with Augmented Pose–Velocity Model**Require:** 
Sampling time $T_{s}$, prediction horizon $N_{p}$, spatial horizon $L_{\mathrm{horiz}}$, identified model $\left(A_{d},B_{d},C_{d},D_{d}\right)$, weights Q⪰0, R≻0, bounds $U_{\min},U_{\max}$, reference path P1:**while** goal not reached **do**2:    Read current state $x_{k}={\left[x_{k},y_{k},\theta_{k}\right]}^{⊤}$3:    Read internal model state $z_{k}={\left[z_{1,k},z_{2,k}\right]}^{⊤}$4:    Find the closest reference point $x_{c}\in P$ to $\left(x_{k},y_{k}\right)$5:    Build the reference horizon ${\left\{x_{i}^{\mathrm{ref}}\right\}}_{i=0}^{N_{p}-1}$ by accumulating arc length from $x_{c}$ up to $L_{\mathrm{horiz}}$ and uniformly resampling it into $N_{p}$ states6:    Form the augmented state $X_{k}={\left[x_{k},y_{k},\theta_{k},z_{1,k},z_{2,k}\right]}^{⊤}$7:   Update the pose-integration matrix $E(\theta_{k})$ using the current orientation $\theta_{k}$8:    Update the augmented model matrices $\left(A_{\mathrm{aug}},B_{\mathrm{aug}}\right)$9:    Construct the lifted prediction matrices (Φ,Γ)10:    Build the local-frame transformations ${\bar{T}}_{i}$ using $\psi_{i}=\theta_{i}^{\mathrm{ref}}$11:    Form $\bar{T}=\mathrm{blkdiag}\left({\bar{T}}_{0},\ldots ,{\bar{T}}_{N_{p}-1}\right)$12:   Build the augmented state-weighting matrix $Q_{\mathrm{aug}}=\mathrm{diag}\left(q_{x},q_{y},q_{\theta },0,0\right)$13:    Build $Q_{\mathrm{big}}=\mathrm{blkdiag}\left(Q_{\mathrm{aug}},\ldots ,Q_{f}\right)$14:    Build the input-weighting matrix $R_{\mathrm{big}}=I_{N_{p}}⊗R$15:    Compute $H=\Gamma^{⊤}{\bar{T}}^{⊤}Q_{\mathrm{big}}\bar{T}\Gamma +R_{\mathrm{big}}$16:    Compute $f=\Gamma^{⊤}{\bar{T}}^{⊤}Q_{\mathrm{big}}\left(\bar{T}\Phi X_{k}-\bar{T}X^{\mathrm{ref}}\right)$17:    Solve $\min_{U}\frac{1}{2}U^{⊤}HU+f^{⊤}U$18:          subject to $U_{\min}\leq U\leq U_{\max}$19:    Apply the first optimal control input $u_{k}^{★}={\left[v_{\mathrm{ref},k}^{★},\omega_{\mathrm{ref},k}^{★}\right]}^{⊤}$20:    Update the internal model state $z_{k+1}=A_{d}z_{k}+B_{d}u_{k}^{★}$

The control formulation relies on a second-order identified discrete-time velocity model with internal states

(17) $$z_{k}={\left[z_{1,k}\;z_{2,k}\right]}^{⊤}\in R^{2},\;z_{k+1}=A_{d}z_{k}+B_{d}u_{k},\;\left[\begin{array}{c} v_{k}\\ \omega_{k} \end{array}\right]=C_{d}z_{k}+D_{d}u_{k},$$

where $u_{k}={\left[v_{\mathrm{ref},k}\;\omega_{\mathrm{ref},k}\right]}^{⊤}$ denotes the reference velocity commands.

The identified matrices $\left(A_{d},B_{d},C_{d},D_{d}\right)$ remain fixed during the MPC experiments because they represent the experimentally identified command-to-velocity dynamics over the operating range considered in this study. Non-ideal effects present in the physical platform, such as actuator dynamics, mechanical asymmetries, friction, and possible wheel-slip effects, are not introduced as explicit physical parameters; instead, their aggregate influence on the measured command-to-velocity response is implicitly reflected in the identified model within the experimental operating range.

It is important to clarify the role of the identified MIMO model within the pose predictor. In the Kinematic MPC, the reference velocity commands are directly used to propagate the robot pose through the kinematic equations. In contrast, the MIMO-MPC first predicts the actual linear and angular velocities from the reference velocity commands using the experimentally identified velocity dynamics. Therefore, the main difference between both predictors lies in whether the command-to-velocity dynamics are explicitly modeled.

The resulting predicted velocities, denoted by $v_{k}$ and $\omega_{k}$, are then integrated through the differential-drive kinematics to predict the three-dimensional robot state

$$x_{k}={\left[x_{k}\;y_{k}\;\theta_{k}\right]}^{⊤}.$$

Although the identified MIMO model provides the two-dimensional velocity vector ${\left[v_{k},\omega_{k}\right]}^{⊤}$, these two velocities determine the evolution of the three pose variables: the linear velocity $v_{k}$ determines the evolution of both Cartesian coordinates $x_{k}$ and $y_{k}$ according to the current robot orientation $\theta_{k}$, while the angular velocity $\omega_{k}$ determines the evolution of $\theta_{k}$. Accordingly, the predicted velocities are mapped into the robot state using the linearized mapping

(18) $$x_{k+1}=x_{k}+E\left(\theta_{k}\right)\left[\begin{array}{c} v_{k}\\ \omega_{k} \end{array}\right],\;E\left(\theta_{k}\right)=T_{s}\left[\begin{array}{cc} \cos\theta_{k} & 0\\ \sin\theta_{k} & 0\\ 0 & 1 \end{array}\right].$$

Defining the augmented state,

(19) $$X_{k}={\left[x_{k}\;y_{k}\;\theta_{k}\;z_{1,k}\;z_{2,k}\right]}^{⊤}\in R^{5},$$

the augmented linear predictor becomes

(20) $$X_{k+1}=A_{\mathrm{aug}}X_{k}+B_{\mathrm{aug}}u_{k},\;A_{\mathrm{aug}}=\left[\begin{array}{cc} I_{3} & E\left(\theta_{k}\right)C_{d}\\ 0 & A_{d} \end{array}\right],\;B_{\mathrm{aug}}=\left[\begin{array}{c} E\left(\theta_{k}\right)D_{d}\\ B_{d} \end{array}\right].$$

Although the identified velocity-model matrices are fixed, the augmented pose-velocity predictor is not constant throughout the experiment. The matrix $E(\theta_{k})$ depends explicitly on the current robot orientation and is recomputed at every sampling instant. Consequently, $A_{\mathrm{aug}}$ and $B_{\mathrm{aug}}$ are also updated at every control iteration. The reference velocities $v_{\mathrm{ref},k}$ and $\omega_{\mathrm{ref},k}$ enter the predictor as optimization inputs through $u_{k}$, rather than as parameters of the identified state-space matrices.

Within the augmented-state penalty, only the pose components are weighted, whereas the internal model states are left unpenalized by defining

(21) $$Q_{\mathrm{aug}}=\mathrm{diag}\left(q_{x},q_{y},q_{\theta },0,0\right)\in R^{5\times 5}.$$

An augmented input-weighting matrix ($R_{\mathrm{aug}}$) is not required because the optimization variable remains $u_{k}\in R^{2}$ (the input dimension is unchanged). Therefore, the same input-weighting matrix $R\in R^{2\times 2}$ is employed, yielding the lifted matrix $R_{\mathrm{big}}=I_{N_{p}}⊗R$.

The QP is constructed using lifted matrices (Φ,Γ) for the augmented model and an extended local-frame transform $\bar{T}=\mathrm{blkdiag}\left({\bar{T}}_{0},\ldots ,{\bar{T}}_{N_{p}-1}\right)$ with

(22) $${\bar{T}}_{i}=\left[\begin{array}{cc} R(\psi_{i}) & 0\\ 0 & I_{3} \end{array}\right]\in R^{5\times 5},$$

which rotates only the planar position components and leaves $\left(\theta ,z_{1},z_{2}\right)$ unchanged. With the augmented reference

(23) $$X_{i}^{\mathrm{ref}}={\left[{\left(x_{i}^{\mathrm{ref}}\right)}^{⊤}\;0\;0\right]}^{⊤}\in R^{5},$$

the matrices of the QP take the same form:

(24) $$H=\Gamma^{⊤}{\bar{T}}^{⊤}Q_{\mathrm{big}}\bar{T}\Gamma +R_{\mathrm{big}},\;f=\Gamma^{⊤}{\bar{T}}^{⊤}Q_{\mathrm{big}}\left(\bar{T}\Phi X_{k}-\bar{T}X^{\mathrm{ref}}\right),$$

where

(25) $$Q_{\mathrm{big}}=\mathrm{blkdiag}\left(Q_{\mathrm{aug}},\ldots ,Q_{f}\right).$$

### 3.4. Experimental Setup and Evaluation

To experimentally evaluate the proposed MPC formulations, a comparative study was conducted using a real differential-drive mobile robot. Two MPC controllers were implemented: a Kinematic MPC and a MIMO-MPC. To ensure a fair comparison, both controllers used the same reference trajectory, sampling period, prediction horizon, spatial horizon, weighting matrices, actuator constraints, and disturbance sequence. Consequently, the predictive model was the only component modified between the two controllers.

The control loop was executed at a sampling frequency of 50Hz, corresponding to a sampling period of $T_{s}=0.02\;s$. A prediction horizon of $N_{p}=15$ was adopted. At each control cycle, a spatial horizon of $L_{\mathrm{horiz}}=1.0\;m$ was extracted from the reference path and uniformly resampled into $N_{p}$ prediction states used by the optimizer. This configuration provided sufficient preview of the reference trajectory while maintaining real-time computational performance on the onboard computer.

The experiments were performed on a closed reference trajectory with an approximate length of 20m. The path combines straight segments with curves of varying curvature, requiring continuous adjustments of both the linear and angular velocities and providing a representative benchmark for evaluating trajectory-tracking performance under realistic navigation conditions. The original trajectory consisted of 2001 sampled points before preprocessing and was spatially resampled with a uniform resolution of 0.01m.

The weighting matrices were empirically tuned through preliminary experimental trials on the real robotic platform to achieve an appropriate compromise between trajectory-tracking accuracy, control effort, and smooth robot motion, while avoiding excessively aggressive control actions. Once a satisfactory closed-loop response was obtained, the selected weights were fixed and remained unchanged throughout all experiments for both controllers to ensure a fair comparison. These matrices represent dimensionless cost weights rather than physical quantities. Increasing a coefficient in the state weighting matrix *Q* increases the penalty associated with the corresponding tracking error, whereas increasing a coefficient in the input weighting matrix *R* penalizes the control effort, leading to smoother but less aggressive control actions.

The selected weighting matrices were

(26) Q=diag(50,400,30),R=diag(10,5),

where the second component of *Q* corresponds to the lateral tracking error. Consequently, the largest weight was assigned to this component to prioritize lateral path-following accuracy while maintaining adequate regulation of the remaining states. These weighting matrices remained unchanged throughout all experiments for both controllers. The remaining experimental parameters are summarized in Table 3.

Two experimental scenarios were considered. The first evaluated both controllers under nominal operating conditions, where the robot tracked the reference trajectory without external disturbances. The second assessed the robustness of both controllers by introducing three predefined perturbations during trajectory execution. Each perturbation consisted of a temporary velocity command applied at predefined locations along the reference path for a specified duration. During each perturbation interval, the control command computed by the MPC was temporarily overridden, and the predefined linear and angular velocity values reported in Table 4 were directly imposed on the robot. Once the specified perturbation duration elapsed, the imposed command was removed and control was returned to the MPC, which then had to recover from the resulting deviation and guide the robot back toward the reference trajectory. This procedure was designed to produce controlled and repeatable deviations while allowing the post-perturbation recovery behavior of both predictive-model formulations to be evaluated under identical conditions.

The externally applied perturbations were not included as known inputs in the predictive model or explicitly incorporated into the MPC objective function. During the perturbation interval, the MPC control action was temporarily overridden as described above. Once control was returned to the MPC, the effect of the perturbation was reflected in the measured robot state and, consequently, in the tracking error used by the controller. Therefore, the disturbed experiments evaluate the inherent recovery capability of each predictive-model formulation rather than the performance of an explicit disturbance-compensation mechanism.

The controllers were evaluated in terms of trajectory-tracking accuracy, robustness, and control smoothness. Trajectory accuracy was quantified using the root mean square error (RMSE), mean absolute error (MAE), 95th percentile error, maximum error, bias, and standard deviation of the lateral tracking error. Robustness was evaluated through the percentage of samples remaining within a ±0.20m lateral error band and the accumulated time spent outside this tolerance. Heading performance was assessed using the RMSE, MAE, and 95th percentile heading error. Finally, control smoothness was quantified through the RMS of the control rate, the RMS jerk of the commanded linear and angular velocities, and the Integrated Smoothness Number (ISN), allowing a quantitative comparison of the aggressiveness of the generated control actions.

## 4. Results

This section presents the experimental comparison between the Kinematic MPC and the MIMO-MPC. Two experimental scenarios were considered. The first evaluates both controllers under nominal operating conditions, whereas the second evaluates their behavior under externally applied perturbations. In both cases, trajectory-tracking accuracy, heading regulation, tracking consistency, and control smoothness were evaluated using the metrics introduced in the previous section.

For visualization purposes, a lateral tolerance band of δ=±0.20m was defined around the reference trajectory. This tolerance was consistently adopted throughout the experimental evaluation to visualize the admissible tracking region, compute the percentage of samples inside the tolerance band, quantify the accumulated time outside the band, and determine the recovery time after each external perturbation.

**Figure 5:**
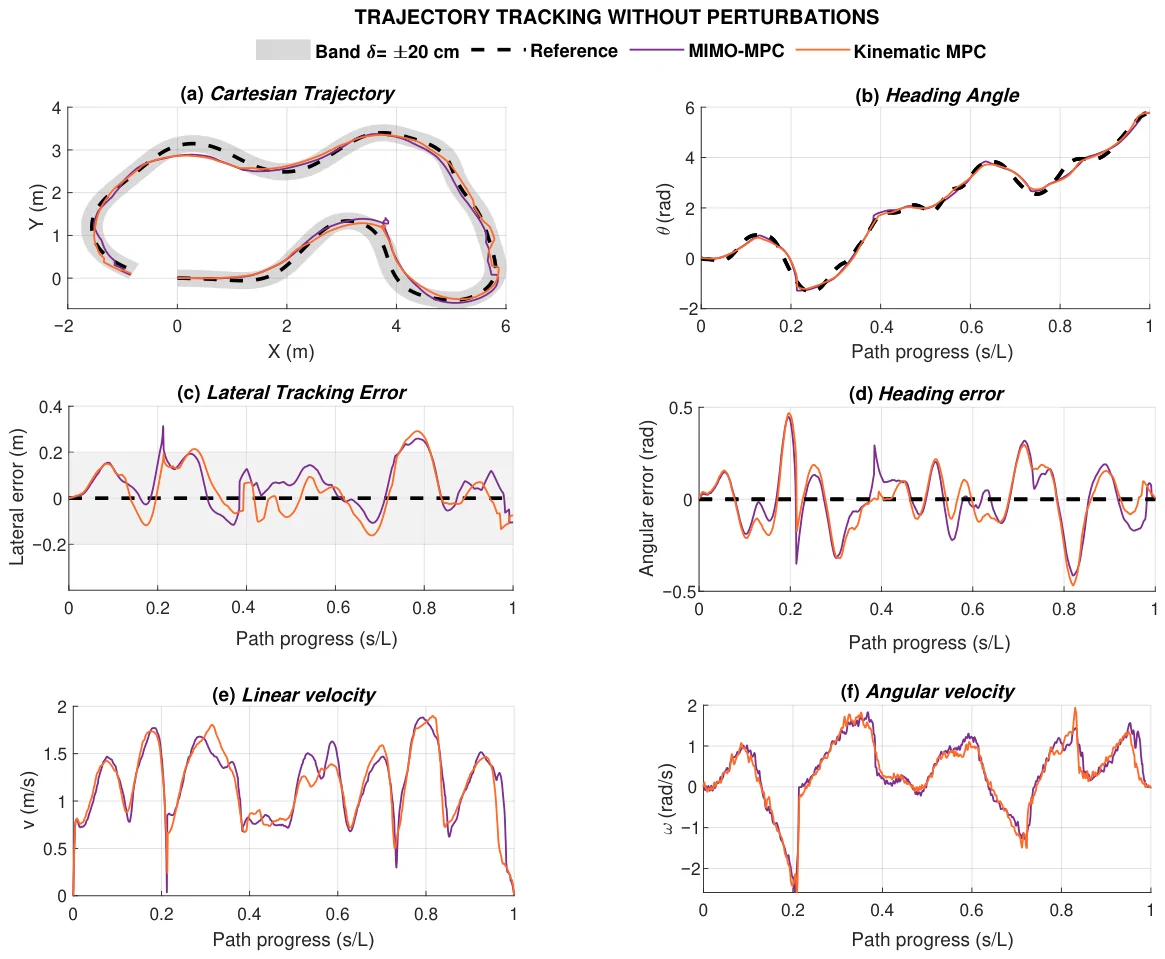
Experimental trajectory-tracking performance under nominal operating conditions. The gray region represents the lateral tolerance band (δ=±0.20 m) around the reference trajectory (black dashed line). The trajectories generated by the MIMO-MPC (purple) and the Kinematic MPC (orange) are compared. The subplots show: (**a**) Cartesian trajectory, (**b**) heading angle as a function of the normalized path progress (s/L), (**c**) lateral tracking error, (**d**) heading error, (**e**) commanded linear velocity, and (**f**) commanded angular velocity.

Figure 5 illustrates the experimental trajectory-tracking performance under nominal operating conditions. The figure presents the Cartesian trajectory together with the lateral tolerance band, the heading evolution along the normalized path progress, the lateral and angular tracking errors, and the commanded linear and angular velocities.

Both controllers successfully complete the reference trajectory while remaining inside the prescribed tolerance band for most of the experiment. The lateral error remains centered around zero for both approaches, whereas localized increases appear mainly in curved sections of the trajectory. Similarly, the heading closely follows the reference orientation, and the commanded velocity profiles evolve continuously throughout the experiment.

The quantitative tracking performance obtained under nominal conditions is summarized in Table 5. The reported metrics provide complementary information regarding the trajectory-following performance. The RMSE and MAE characterize the average lateral tracking accuracy, whereas the 95th percentile and maximum error describe larger deviations occurring during the experiment. The bias indicates the mean lateral offset with respect to the reference trajectory, while the standard deviation measures the variability of the tracking error. Likewise, the heading metrics quantify the orientation regulation throughout the trajectory.

**Figure 6:**
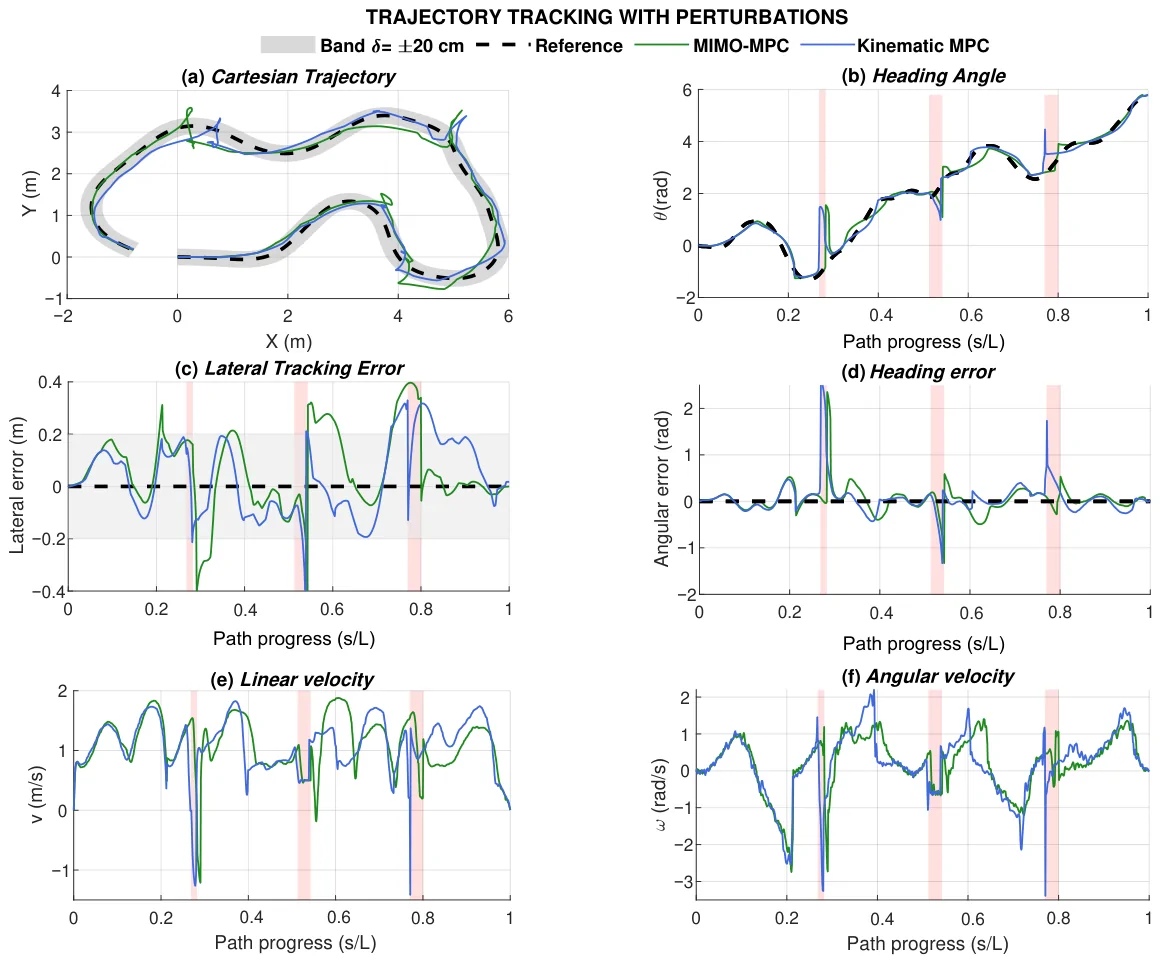
Experimental trajectory-tracking performance under external perturbations. The gray shaded region represents the lateral tolerance band (δ=±0.20 m) around the reference trajectory (black dashed line). The trajectories generated by the MIMO-MPC (green) and the Kinematic MPC (blue) are shown. The red shaded regions indicate the time intervals during which the external perturbations were applied. The subplots show: (**a**) Cartesian trajectory, (**b**) heading angle as a function of the normalized path progress (s/L), (**c**) lateral tracking error, (**d**) heading error, (**e**) commanded linear velocity, and (**f**) commanded angular velocity.

Figure 6 presents the experimental results obtained when three predefined perturbations (P1–P2–P3) are introduced during trajectory execution. The highlighted disturbance intervals correspond to the periods during which the MPC-generated control commands were temporarily overridden by the predefined velocity commands reported in Table 4; immediately after each interval, control was returned to the corresponding MPC. The perturbations temporarily increase the lateral and angular tracking errors. After each disturbance, both controllers generate corrective actions that progressively return the robot toward the reference trajectory. The quantitative performance obtained during the disturbed experiments is summarized in Table 6. Compared with the nominal experiments, the disturbance scenario produces larger tracking errors, reflecting the transient deviations introduced by the external velocity perturbations. The tracking metrics quantify these deviations throughout the complete experiment, whereas the heading metrics describe the orientation response during both the perturbation and recovery phases.

The control-smoothness metrics exhibit different trends between the nominal and perturbed operating conditions. Under nominal conditions, the MIMO-MPC shows higher RMS control-rate and RMS jerk values than the Kinematic MPC for both the linear and angular velocity commands. Under external perturbations, the opposite trend is observed, with the MIMO-MPC exhibiting lower RMS control rate and RMS jerk values in both channels. Regarding tracking consistency, the Kinematic MPC maintains a higher percentage of samples within the prescribed lateral tolerance band and a shorter accumulated time outside the band under both operating conditions. The ISN values remain similar for both controllers in the nominal and perturbed experiments.

The overall tracking consistency together with the smoothness of the generated control actions are summarized in Table 7.

The first two metrics describe the global tracking consistency with respect to the prescribed lateral tolerance band of δ=±0.20m. The remaining metrics characterize the temporal variation of the generated control commands. The RMS control rate measures the average variation im the commanded velocities, whereas the RMS jerk quantifies the smoothness of the corresponding acceleration profiles. The Integrated Smoothness Number (ISN) provides a global indicator of the overall smoothness of the generated control actions.

Finally, the transient recovery following each perturbation was evaluated using the same lateral tolerance band adopted throughout the experimental analysis. Recovery time was defined as the elapsed time from the end of each perturbation until the lateral tracking error satisfied

(27) $$|e_{y}|\leq \delta ,$$

with δ=0.20m, and remained inside this tolerance for at least 0.5s. If the robot already satisfied this condition at the end of the perturbation, the recovery time was considered zero. The resulting recovery times for each perturbation are summarized in Table 8.

Unlike the global tracking metrics presented in the previous tables, the recovery time specifically characterizes the transient behavior immediately after each perturbation. Therefore, it complements the overall trajectory-tracking analysis by quantifying the time required for the robot to re-enter the prescribed lateral tracking tolerance band after the external disturbance has ceased.

## 5. Discussion

The experimental results show that the influence of the predictive model depends strongly on the operating conditions. Since both controllers used the same MPC formulation, constraints, weighting matrices, prediction horizon, reference trajectory, and disturbance sequence, the observed differences can primarily be attributed to the predictive model employed by each controller. This comparison follows the fundamental MPC principle described by Mayne et al. [1], according to which closed-loop performance depends on the ability of the prediction model to adequately represent the controlled system over the finite horizon.

Under nominal conditions, the conventional Kinematic MPC achieved slightly better overall trajectory-tracking accuracy. Its lateral RMSE was 0.1031m, compared with 0.1187m for the MIMO-MPC, corresponding to a reduction of approximately 13.1%. The lateral MAE was also reduced from 0.0924m to 0.0818m, while the percentage of samples within the ±0.20m tolerance band increased from 90.98% to 93.23%. These results indicate that the kinematic model is sufficiently accurate when the robot remains close to its nominal operating regime. This is consistent with Yang et al. [9], who showed that a linearized kinematic tracking-error model can provide accurate and smooth trajectory tracking when combined with appropriate input and input-increment constraints. Therefore, increasing the predictive-model complexity does not necessarily improve nominal tracking when the dominant behavior is already captured by the simpler model.

A different trend was observed under external perturbations. Although the Kinematic MPC retained a lower global lateral RMSE, with 0.1726m compared with 0.2065m for the MIMO-MPC, the MIMO-MPC produced a better angular and transient response. The heading RMSE decreased from 0.7617rad to 0.6503rad, representing an improvement of approximately 14.6%. Moreover, the recovery time following P2 was reduced from 1.32s to 0.85s, whereas after P3 it decreased from 1.98s to 1.50s. These reductions correspond to approximately 35.6% and 24.2%, respectively. Thus, the main benefit of the MIMO-MPC is not a reduction in the average lateral error, but a faster restoration of the robot orientation and trajectory after substantial deviations.

This result is consistent with the disturbance-rejection MPC framework developed by Sun et al. [11], in which disturbance information incorporated into the predictive controller improved the tracking behavior of a wheeled mobile robot subjected to matched disturbances. Sun et al. [12] similarly demonstrated that nominal predictions alone may be insufficient when additive disturbances cause the real system to depart from the predicted trajectory, motivating robust predictive formulations. More recently, Li et al. [13] experimentally showed that combining MPC with disturbance estimation improves trajectory tracking in the presence of unknown disturbances. Although the present approach does not explicitly estimate the applied perturbations, the experimentally identified MIMO state-space model appears to encode part of the actuator and robot response that is absent from the ideal kinematic equations, thereby improving the prediction of the post-disturbance transient.

The control-smoothness results further support this interpretation. An interesting result is the different trend observed between nominal and perturbed operating conditions. Under nominal conditions, the Kinematic MPC exhibited lower control-rate and jerk values than the MIMO-MPC, indicating that the simpler kinematic predictor generated smoother control actions when the robot remained close to its nominal operating regime. Under disturbed conditions, however, this trend was changed. The angular-velocity RateRMS decreased from 7.619 for the Kinematic MPC to 5.737 for the MIMO-MPC, corresponding to a reduction of approximately 24.7%. Similarly, the angular JerkRMS decreased from 524.22 to 394.42, a reduction of approximately 24.8%. The linear RateRMS and JerkRMS were also reduced by approximately 15.7% and 16.1%, respectively. This change in trend suggests that the influence of the identified command-to-velocity dynamics becomes more relevant during the transient response to external perturbations. While the Kinematic MPC produces smoother control actions under nominal operation, the MIMO-MPC recovers from the perturbations using less abrupt variations in the commanded velocities. This behavior is relevant in real robots because aggressive command variations may increase actuator stress, wheel slip, mechanical vibration, and measurement noise. The importance of jointly considering tracking accuracy and control smoothness was also emphasized by Yang et al. [9], while Wang et al. [14] showed that augmenting MPC with a disturbance observer can improve robustness without violating the physical constraints of the mobile platform.

The results are also aligned with recent data-driven predictive-control studies. Tang et al. [6] used a deep Koopman representation to construct a predictive model of a wheeled mobile robot and reported a 46.03% reduction in tracking error relative to their conventional MPC benchmark when the learned model was combined with disturbance estimation and adaptive prediction. Direct numerical comparison with that result is not appropriate because the robot, trajectory, disturbance model, and evaluation criteria differ from those used in the present study. Nevertheless, both studies support the general conclusion that improving the predictive model can be particularly beneficial when uncertain or unmodeled dynamic effects become relevant.

The faster recovery observed here also complements the experimental results of Guffanti et al. [15], who introduced a dynamic look-ahead re-entry mechanism to assist a differential-drive robot after it exited a prescribed lateral tolerance band. Their work showed that explicit recovery mechanisms can improve robustness when deviations become too large for nominal MPC tracking. In contrast, the present results demonstrate that part of this improvement can also be obtained by modifying only the predictive model: without changing the optimization framework or adding a dedicated re-entry controller, the MIMO-MPC reduced the recovery time after the two most demanding perturbations.

Overall, neither controller can be considered universally superior. The conventional Kinematic MPC provides better average lateral accuracy under nominal conditions and remains competitive under perturbations. The MIMO-MPC, however, provides better heading regulation, faster recovery after the largest disturbances, and smoother control commands. Therefore, the selection of the predictive model should depend on the requirements of the application. A Kinematic MPC is attractive for structured environments in which nominal tracking accuracy and model simplicity are the main priorities. Conversely, the MIMO-MPC is advantageous when the robot is expected to encounter external perturbations, actuator dynamics, surface-dependent effects, or other behaviors that are not adequately represented by ideal kinematic equations.

The findings of this study should be interpreted within the scope of the evaluated experimental conditions. The comparison was conducted using two predictive-model formulations implemented on a single robotic platform and validated over a representative trajectory with controlled external perturbations. Although these conditions provide a consistent basis for comparing both predictive models, future studies may further investigate the observed behavior using additional robotic platforms and operating scenarios.

## 6. Conclusions

This study presented an experimental comparison between a Kinematic MPC and a MIMO-MPC implemented on a four-wheel differential-drive mobile robot. The MIMO-MPC employs an experimentally identified MIMO state-space model as its predictive model, whereas the Kinematic MPC uses a conventional kinematic model. Both controllers were evaluated under identical operating conditions through real-world experiments conducted under nominal and externally perturbed operating conditions.

The experimental results demonstrated that the influence of the predictive model depends on the operating conditions. Under nominal conditions, the Kinematic MPC achieved the best overall lateral tracking performance, obtaining a lateral RMSE of 0.1031 m and maintaining 93.23% of the trajectory within the ±0.20 m tolerance band. Under externally perturbed conditions, the MIMO-MPC exhibited superior transient behavior by reducing the heading RMSE from 0.7617 rad to 0.6503 rad, shortening the recovery time after the largest disturbances by up to 35.6%, and producing smoother control actions, with reductions of approximately 24.7% in angular RateRMS and 24.8% in angular JerkRMS.

These findings demonstrate that improving the predictive model does not necessarily lead to better nominal trajectory-tracking accuracy. Instead, its principal contribution lies in enhancing disturbance recovery and producing smoother control actions when the robot operates under non-ideal conditions. Consequently, the selection of the predictive model should be guided by the expected operating conditions and the specific performance requirements of the application rather than by nominal tracking accuracy alone.

The present study was limited to the experimental comparison of two predictive-model formulations implemented on a single robotic platform under controlled operating conditions. Nevertheless, the reported results provide valuable experimental evidence of how predictive-model choice influences the closed-loop performance of MPC under the evaluated scenarios.

Future work will expand this comparative analysis by incorporating adaptive and learning-based predictive models, as well as by validating the proposed methodology in more challenging navigation scenarios involving varying terrain conditions, payload changes, and dynamic environments. In addition, future studies will investigate the repeatability of the identified model across independent experimental sessions and alternative excitation profiles specifically designed for system identification.

## Figures and Tables

**Table 1:** Main specifications of the mobile robotic platform.

Component/Parameter	Specification
Mobile platform	Four-wheel differential-drive robot
Chassis	Recon Chassis Kit
Total mass	Approx. 3.5kg
Motors	4× 5203 Series Yellow Jacket
Gear ratio	19.2:1
Nominal motor output speed	312RPM
Motor feedback	Integrated quadrature encoders (3.3–5 V)
Motor controllers	2× dual-channel ROBOCLAW
Maximum driver current	15A per channel
Maximum linear velocity	Approx. 1.5m/s
Maximum angular velocity	Approx. 1.75rad/s
IMU	WitMotion WT901C-TTL
Environment sensing	2D LiDAR
Microcontroller	STM32 NUCLEO-F207ZG
Motor communication	USART, 38,400 baud
Onboard computer	Intel NUC BNUC11TNKV70002
Processor	Intel Core i7-1185G7 (11th generation)
Operating system	Ubuntu 20.04
Middleware	ROS 2 Foxy Fitzroy
Navigation software	Nav2 and SLAM Toolbox
Sampling frequency	50Hz
Sampling period	$T_{s}=0.0202\;s$

**Table 2:** Comparison of identified MIMO models with different orders using validation data.

Order	${\mathrm{FIT}}_{v}$ (%)	${\mathrm{RMSE}}_{v}$ (m/s)	${\mathrm{FIT}}_{\omega }$ (%)	${\mathrm{RMSE}}_{\omega }$ (rad/s)
1	96.51	0.0386	−0.84	0.5816
2	97.25	0.0304	87.72	0.0708
3	97.43	0.0284	87.79	0.0704
4	97.42	0.0285	88.04	0.0690
5	97.41	0.0287	85.23	0.0852

**Table 3:** Experimental setup used for the comparative evaluation.

Parameter	Value
Control frequency	50Hz
Sampling period, $T_{s}$	0.02s
Prediction horizon, $N_{p}$	15
Spatial horizon, $L_{\mathrm{horiz}}$	1.0m
Reference path resolution	0.01m
State weighting matrix	Q=diag(50,400,30)
Input weighting matrix	R=diag(10,5)
Linear velocity bounds	±2.0m/s
Angular velocity bounds	±2.0rad/s
State estimation	Filtered odometry
Optimization solver	Operator Splitting Quadratic Program (OSQP)

**Table 4:** External perturbations applied during the disturbed experiments.

Perturbation	Path Index	v (m/s)	ω (rad/s)	Duration (s)
P1	500	0.0	0.75	3.0
P2	1000	0.5	–0.50	2.0
P3	1500	0.2	1.00	2.0

**Table 5:** Trajectory-tracking performance under nominal operating conditions.

Metric	MIMO-MPC	Kinematic MPC
RMSE lateral (m)	0.1187	0.1031
MAE lateral (m)	0.0924	0.0818
95th percentile lateral (m)	0.2474	0.2138
Maximum lateral error (m)	0.3631	0.2915
Bias (m)	0.0435	0.0146
Standard deviation (m)	0.1105	0.1021
RMSE heading (rad)	0.1450	0.1337
MAE heading (rad)	0.1076	0.0995
95th percentile heading (rad)	0.3189	0.2828

**Table 6:** Trajectory-tracking performance under external perturbations.

Metric	MIMO-MPC	Kinematic MPC
RMSE lateral (m)	0.2065	0.1726
MAE lateral (m)	0.1426	0.1341
95th percentile lateral (m)	0.4336	0.3405
Maximum lateral error (m)	0.6428	0.6009
Bias (m)	0.0361	0.0239
Standard deviation (m)	0.2034	0.1710
RMSE heading (rad)	0.6503	0.7617
MAE heading (rad)	0.3784	0.4341
95th percentile heading (rad)	1.5340	1.9858

**Table 7:** Tracking consistency and control smoothness.

Metric	Nom.	Nom.	Pert.	Pert.
	**MIMO**	**Kin.**	**MIMO**	**Kin.**
Samples inside band (%)	90.98	93.23	71.92	80.40
Time outside band (s)	2.16	1.56	9.32	6.31
RMS control rate (lin.)	2.190	1.883	5.718	6.785
RMS control rate (ang.)	1.408	1.288	5.737	7.619
RMS jerk (lin.)	127.92	100.91	381.25	454.17
RMS jerk (ang.)	82.73	64.50	394.42	524.22
ISN	0.97923	0.97991	0.97244	0.97145

**Table 8:** Recovery time to the ±0.20 m lateral tracking tolerance band.

Perturbation	MIMO-MPC (s)	Kinematic MPC (s)
P1	0.00	0.00
P2	0.85	1.32
P3	1.50	1.98

## Data Availability

Dataset available on request from the authors.

## Abbreviations

MPC	Model Predictive Control
MIMO	Multiple-Input Multiple-Output
DDMR	Differential-Drive Mobile Robot
ROS 2	Robot Operating System 2
IMU	Inertial Measurement Unit
LiDAR	Light Detection and Ranging
MCU	Microcontroller Unit
CPU	Central Processing Unit
USART	Universal Synchronous/Asynchronous Receiver/Transmitter
USB	Universal Serial Bus
RC	Radio Control
FIT	Model Fit Percentage
RMSE	Root Mean Square Error
MAE	Mean Absolute Error
RMS	Root Mean Square
QP	Quadratic Programming
OSQP	Operator Splitting Quadratic Program
ISN	Integrated Smoothness Number

## References

[1] Mayne D.Q., Rawlings J.B., Rao C.V., Scokaert P.O.M. (2000). Constrained Model Predictive Control: Stability and Optimality. Automatica.

[2] Xu C., Zhou X., Chen R., Li B., He W., Li Y., Ye F. (2025). Trajectory Tracking for 3-Wheeled Independent Drive and Steering Mobile Robot Based on Dynamic Model Predictive Control. Appl. Sci..

[3] Ali A.M., Shen C., Hashim H.A. (2024). A Linear MPC with Control Barrier Functions for Differential Drive Robots. IET Control Theory Appl..

[4] Abed A.M., Rashid Z.N., Abedi F., Zeebaree S.R., Sahib M.A., Mohamad Jawad A.J., Redha Ibraheem G.A., Maher R.A., Abdulkareem A.I., Ibraheem I.K. (2022). Trajectory tracking of differential drive mobile robots using fractional-order proportional-integral-derivative controller design tuned by an enhanced fruit fly optimization. Meas. Control.

[5] Zhang K., Wang J., Xin X., Li X., Sun C., Huang J., Kong W. (2022). A Survey on Learning-Based Model Predictive Control: Toward Path Tracking Control of Mobile Platforms. Appl. Sci..

[6] Tang M., Zhang Y., Yu S., Li J., Tang K. (2025). Trajectory Tracking Model Predictive Control for Mobile Robot Based on Deep Koopman Operator Modeling. Robot. Auton. Syst..

[7] Bouzoualegh S., Guechi E.H., Kelaiaia R. (2018). Model Predictive Control of a Differential-Drive Mobile Robot. Acta Univ. Sapientiae Electr. Mech. Eng..

[8] Mondal K., Rodriguez A.A., Manne S.S., Das N., Wallace B. Comparison of Kinematic and Dynamic Model Based Linear Model Predictive Control of Non-Holonomic Robot for Trajectory Tracking: Critical Trade-offs Addressed. Proceedings of the American Control Conference.

[9] Yang H., Guo M., Xia Y., Cheng L. (2018). Trajectory Tracking for Wheeled Mobile Robots via Model Predictive Control with Softening Constraints. IET Control Theory Appl..

[10] Falcone P., Borrelli F., Asgari J., Tseng H.E., Hrovat D. (2007). Predictive Active Steering Control for Autonomous Vehicle Systems. IEEE Trans. Control Syst. Technol..

[11] Sun Z., Xia Y., Dai L., Liu K., Ma D. (2017). Disturbance Rejection MPC for Tracking of Wheeled Mobile Robot. IEEE/ASME Trans. Mechatron..

[12] Sun Z., Dai L., Liu K., Xia Y., Johansson K.H. (2018). Robust MPC for Tracking Constrained Unicycle Robots with Additive Disturbances. Automatica.

[13] Li P., Yang H., Wang S. (2023). Model Predictive Tracking Control with Disturbance Compensation for Wheeled Mobile Robots in an Environment with Obstacles. J. Frankl. Inst..

[14] Wang D., Gao Y., Wei W., Yu Q., Wei Y., Li W., Fan Z. (2024). Sliding Mode Observer-Based Model Predictive Tracking Control for Mecanum-Wheeled Mobile Robot. ISA Trans..

[15] Guffanti D., Mora Murillo M.F., Bustamante Sanchez S., Obregón Gutiérrez J.O., Hinojosa M.A., Brunete A., Hernando M., Álvarez D. (2026). Robust Model Predictive Control with a Dynamic Look-Ahead Re-Entry Strategy for Trajectory Tracking of Differential-Drive Robots. Sensors.

